# Beneficial Effect of Immune-Enhanced Enteral Nutrition on Immune Function in Patients With Severe Neurological Diseases: A Single-Center Randomized Controlled Trial

**DOI:** 10.3389/fnut.2021.685422

**Published:** 2021-08-23

**Authors:** Ke Chao, Dong Wang, Hongfu Yang, Ning Ma, Qilong Liu, Xiaoge Sun, Rongqing Sun

**Affiliations:** ^1^Extracardiac Care Unit, Henan Provincial Chest Hospital, Zhengzhou, China; ^2^Intensive Care Unit, The First Affiliated Hospital of Zhengzhou University, Zhengzhou, China

**Keywords:** critical illness, nutritional status (NPK), cellular immunity, enteral nutrition, neurologic disease, randomized controlled trial

## Abstract

**Background:** Undernutrition is the main reason for the use of artificial nutrition in patients with severe neurological diseases. However, the clinical and immunological outcomes of enteral nutrition supplemented with immunomodulatory nutrients in these patients remain unclear.

**Methods:** In this single-center, randomized controlled study, 57 patients with severe neurological diseases were randomly divided into the following two groups according to the type of enteral nutrition they would receive: immune-enhancing (IE) (*n* = 27) and non-IE (NIE) (*n* = 30). The IE and NIE groups received enteral nutrition supplemented with immunomodulatory nutrients and standard enteral nutrition, respectively. We compared the nutritional status and the state of cellular immunity between the patients of the two groups. Clinical and immunological variables were evaluated following nutritional treatment.

**Results:** Feeding intolerance was lower in the IE than that in the NIE group (*P* = 0.04). However, there were no significant differences between the results of the two groups in terms of length of stay in the intensive care unit or hospital, extubation time, or body mass index (*P* > 0.05). The CD4+ T-lymphocyte count and CD4^+^/CD8+ ratio in the peripheral blood increased significantly in the IE group. The expression of CD28 activated cell surface markers was higher in the IE than in the NIE group. In addition, increased plasma interferon-γ levels were recorded in the IE group, whereas the levels of tumor necrosis factor-α (TNF-α), interleukin (IL)-6, IL-8, and IL-10 decreased.

**Conclusions:** Immune-enhanced enteral nutrition could improve the immune status and feeding tolerance in patients with severe neurological diseases.

**Trial Registration:**www.chictr.org.cn/index.aspx, identifier: ChiCTR-IPR-17013909.

## Introduction

Due to stress response, critically ill patients have a high metabolic state, often accompanied by disturbances in consciousness and swallowing dysfunction, which directly affect the intake and utilization of nutrients, leading to malnutrition (1, 2). In patients with critical illness, malnutrition is associated with increased incidence of infection and longer hospital stay (3). When the intestinal function is maintained, enteral nutrition is the preferred feeding method (4).

Immune dysfunction, respiratory muscle weakness, and decreased ventilation capacity and gastrointestinal tolerance are common issues in patients hospitalized in the intensive care unit (ICU) (5). Laboratory and clinical studies have shown that the addition of immune-enhancing nutrients, such as arginine, glutamine, nucleotides, omega-3 fatty acids, L-carnitine, and taurine, modulate the pathophysiology of critical diseases, such as inflammation, oxidative stress response, and impaired immune function (6–8).

Several meta-analyses have reported that the addition of immunomodulatory nutrients to the enteral nutrition could significantly reduce the incidence of infectious diseases and promote the recovery from critical illnesses compared with the standard enteral nutrition (9). The Society of Critical Care Medicine and the American Society for Parenteral and Enteral Nutrition guidelines state that artificial nutrition should be used for specific patients, including those critically ill requiring respiratory assistance, those with severe sepsis, those recommended to undergo surgical procedures, and those hospitalized in the ICU (10).

In recent years, the influence of systemic immune status on the prognosis of patients with severe neurological diseases, such as cerebral hemorrhage, stroke, craniocerebral trauma, and hypoxic-ischemic encephalopathy, has received increased attention (11–13). Systemic immunosuppression of patients with traumatic brain injury leads to an increased risk of nosocomial infections, which is associated with an increased risk of death, prolonged hospital stay, and neurological dysfunction (14–16). Stroke-induced damage to the central nervous system leads to secondary immunodeficiency and infection, with the latter being the main cause of death in patients with stroke (17, 18).

Clinicians are increasingly paying attention to the issue of artificial nutrition (19). Moreover, few researchers have studied the clinical and immunological mechanisms of artificial nutrition in patients with severe neurological diseases (20). In the present trial, patients with severe neurological diseases, such as cerebral hemorrhage, stroke, craniocerebral trauma, and hypoxic-ischemic encephalopathy, were given enteral nutrition supplemented with immunomodulatory nutrients or standard enteral nutrition, and the effects of the two were compared to determine the most effective type of nutrition for patients with severe neurological diseases.

## Materials and Methods

### Study Design

We initially evaluated 77 patients with severe neurological diseases treated in the Surgical ICU of the First Affiliated Hospital of Zhengzhou University, Henan Province, China, between February and December 2018. The inclusion criteria were as follows: (a) age ≥ 18 years, (b) Acute Physiology and Chronic Health Evaluation II (APACHE II) score ≥ 10 points, (c) estimated clinical enteral nutritional support required for ≥ 5 days, (d) diagnosis of a neurological disease on first admission (e.g., traumatic brain injury, cerebral hemorrhage, cerebral infarction, hypoxic-ischemic encephalopathy), (e) Glasgow Coma Scale score ≤ 12 points, and (f) neurological diseases diagnosed during the ICU stay or admitted to the ICU within 24 h of onset. The research protocol was approved by the Scientific and Clinical Ethics Committee of the First Affiliated Hospital of Zhengzhou University (science-2017-LW-12). It has been registered in the China Clinical Trial Center under the registration number ChiCTR-IPR-17013909.

**Figure 1:**
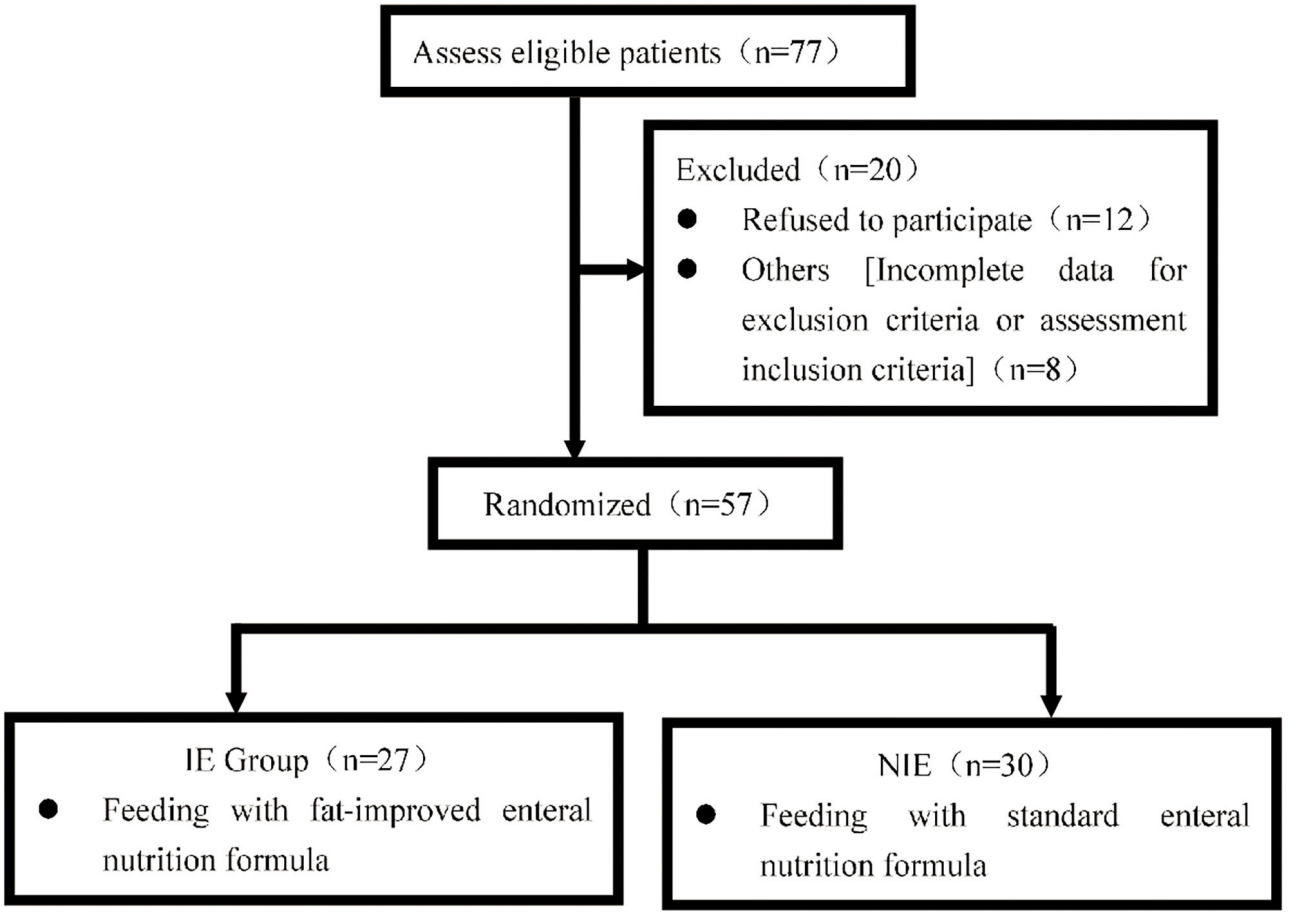
Patients were approached and recruited according to the inclusion and exclusion criteria. 419 NIE, non-immune-enhancing; IE, immune-enhancing.

Eight patients did not meet the inclusion criteria and 12 refused to participate (Figure 1); therefore, 57 patients were finally included. The included patients had severe neurological diseases, including cerebral hemorrhage (33.3%), cerebral infarction (5.3%), traumatic brain injury (59.6%), and ischemic hypoxic encephalopathy (1.8%).

The study was conducted in accordance with the Declaration of Helsinki and written informed consent was obtained from all patients before the start of the study (Figure 1).

### Nutrition Support

Before implementing enteral nutrition, computer-generated random numbers were used to assign patients to the immune-enhancing (IE) (*n* = 27) or the non-IE (NIE) (*n* = 30) group. The IE and the NIE groups received artificial nutrition supplemented with taurine and standard enteral nutrition, respectively. The 57 patients in the Surgical ICU were randomly assigned on a 1:1 basis. Researchers and clinicians were blinded to treatment allocation.

Patients were administered the IE enteral nutrition formula (TPF-FOS; Abbott Laboratories, Abbott Park, IL, USA), which contains taurine, L-carnitine, and medium chain triglycerides, or the standard enteral formula (TPF-TP; Abbott Laboratories) (Supplementary Table 1) depending on their group assignment within 24–48 h after admission. The enteral nutrition formula was masked from the personnel administering the feedings; similarly, they were blind to clinical and laboratory results. Enteral nutrition was administered through a nasogastric tube.

Specialized personnel calculated the caloric value required by each patient according to their standard body weight [ideal body weight (kg) = height (cm) - 105]. Dose and speed were adjusted according to the simple gastrointestinal function scoring method (Supplementary Table 2). Enteral nutrition was initially evaluated every 4–6 h, and enteral nutrition infusion was adjusted according to the score, as follows: (1) Total score 0–2: continued enteral nutrition, increased or maintained original speed, symptomatic treatment; (2) Total score 3–4: continued enteral nutrition, speed reduction, and re-evaluation after 2 h; (3) Total score ≥ 5 points: pause of the enteral nutrition and performance of the corresponding treatment (including the use of prokinetic drugs, changing enteral nutrition infusion route, etc.); re-evaluation every 4–6 h after adjustment; when the infusion remained stable, there was no need for readjustment and it could be evaluated once a day. The dosage was usually increased with the starting dose as a gradient; when the starting speed was 25 ml/h, it was increased by 25 ml/h each time. The reduction was usually based on the initial dose as a gradient decrease; when the initial speed was 10 ml/h, the reduction was 10 ml/h. A minimal dose of enteral feeding (500 mL) was applied on day 1 and then continued on days 2–5. When the patient developed feeding intolerance, enteral nutrition was suspended and the patient was treated accordingly (including the use of prokinetic drugs, replacement of enteral nutrition infusion routes, etc.). Feeding intolerance was defined as discontinuation due to gastrointestinal issues (e.g., severe bloating, diarrhea, vomiting, residual gastric volume ≥ 300 ml within 6 h, or subjective discomfort).

### Data Collection

We collected the baseline characteristics of population data, clinical diagnosis, and disease severity indicators, such as the APACHE II score and surgical status. We recorded the enteral nutrition time (in days); feeding intolerance; drugs administered, such as sedatives, vasoactive drugs, and gastrointestinal motility drugs; day of ICU admission; study day 6; liver function before discharge; blood levels of C-reactive protein and procalcitonin; and clinical outcome indicators, such as the mechanical ventilation time, ICU stay period, and hospitalization period (in days).

### Cytokines

On day 6, the peripheral blood of the patients was collected in anti-coagulation tubes containing ethylenediaminetetraacetic acid, and plasma was obtained by centrifugation of the blood at 4°C. The concentrations of tumor necrosis factor-α (TNF-α), interleukin (IL)-8, IL-6, IL-1β, IL-10, and IFN-γ in the plasma were determined using ELISA (R&D Systems Inc., Minneapolis, MN, USA) assays.

### Flow Cytometry Analysis

Fresh human mononuclear cells were isolated from the peripheral blood using density gradient centrifugation. To identify the cell surface phenotype, the cells were incubated with a primary antibody conjugated to a fluorescent dye. Viable cells (1 × 10^5^) were stained with anti-human CD3, CD8, CD4, CD28, Tim3, and PD-1 antibodies. Dead cells were stained using 7-AAD. Isotype controls were performed on each cell type. The cells were analyzed using flow cytometry (CantoII, BD Biosciences, San Jose, CA, USA) and Diva analysis software (BD Biosciences). During the analysis, the percentage of positive cells was calculated.

### Statistical Analysis

Statistical analysis of the data was performed using SPSS 17.0 (SPSS, Inc., IBM, Chicago, IL, USA) and GraphPad Prism 5.0 (GraphPad Software, La Jolla, CA, USA). The data were analyzed using normal tests for continuous variables. The χ^2^, rank sum, *t*-, and rank comparison tests were used for qualitative data of disordered classification, qualitative data of ordered classification, quantitative data that matched the normal distribution, and comparison analyses, respectively. The statistically significant level was set at *P* < 0.05.

## Results

### General and Nutritional Status

The characteristics of patients included in the trial and the data comparison between the NIE and IE groups are shown in Table 1. There were no significant differences in age, sex, weight, body mass index, surgical status, disease composition, and disease severity (APACHE II score) between the two groups of patients (*P* > 0.05).

**Table 1:** Demographic data and parameters on admission.

**Parameter**		**IE group (*n* = 27)**	**NIE group (*n* = 30)**	***P-*value**
Sex	Men	22	22	0.46
	Women	5	8	
	Age (yr)	47.52 ± 16.24	52.57 ± 13.38	0.20
	Weight (kg)	62.88 ± 7.32	64.01 ± 7.52	0.52
	BMI (kg/m^2^)	22.23 ± 2.24	22.43 ± 2.58	0.72
Admission diagnosis	Cerebral hemorrhage	6	13	0.24
	Stroke	1	2	
	Craniocerebral trauma	19	15	
	Hypoxic-ischemic encephalopathy	1	0	
Complications	Hypertension	7	15	0.06
	Coronary heart disease	0	3	0.09
	Diabetes	2	0	0.13
	Surgical operation	11	17	0.23
	APACHE II	19.63 ± 4.87	19.47 ± 5.79	0.91
	Enteral nutrition time (d)	17.44 ± 6.28	19.13 ± 9.69	0.44

*Values are presented as mean ± standard deviation*.

*BMI, Body mass index; APACHE II, Acute physiology and chronic health evaluation II; NIE, non-immune-enhancing; IE, immune-enhancing*.

### Immunologic Responses After Nutrition

The CD4^+^ T-lymphocyte count and the CD4^+^/CD8^+^ ratio on day 6 were higher in the IE than those in the NIE group, whereas the ratio of CD8^+^ T-lymphocytes was not significantly different. Similarly, the proportions of CD28 co-stimulatory surface marker were higher in the IE than those in the NIE group. There was no difference in the expression of the co-inhibitory molecules, PD-1 and TIM-3, between the two groups. The immune system function was significantly enhanced after IE feeding (Table 2).

**Table 2:** Changes of immune indicators in the NIE and IE groups.

**Parameter**	**IE group**	**NIE group**	***P*-value**
CD4^+^,%	26.76 ± 2.24	20.36 ± 2.00	0.04
CD8^+^,%	15.66 ± 1.35	15.23 ± 1.35	0.82
CD4^+^/CD8^+^	1.94 ± 0.20	1.32 ± 0.12	0.01
CD4^+^CD28^+^	77.44 ± 2.82	69.57 ± 2.57	0.04
CD4^+^Tim3^+^	21.1 ± 1.66	21.06 ± 2.31	0.98
CD4^+^PD-1^+^	21.22 ± 2.633	15.75 ± 2.21	0.11
CD8^+^CD28^+^	64.15 ± 4.03	51.05 ± 4.87	0.04
CD8^+^Tim3^+^	15.96 ± 1.85	15.29 ± 2.08	0.80
CD8^+^PD-1^+^	16.28 ± 1.77	16.17 ± 2.06	0.96

*Values are presented as mean ± standard deviation*.

*NIE, non-immune-enhancing; IE, immune-enhancing*.

### Cytokine Response

As shown in Table 3, the expression of IFN-γ was higher in the IE than that in the NIE group (*P* < 0.05) on day 6 after admission. In contrast, the levels of IL-6, IL-8, IL-10, IL-1β, and TNF-α were lower in the IE than those in the NIE group (*P* < 0.05). Moreover, higher ratios of IL-6/IL-10, TNF-α/IL-10, IL-8/IL-10, and IL-1β/IL-10 were observed in the NIE group (*P* < 0.05). These results indicated that the inflammatory state was lower in the IE than that in the NIE group.

**Table 3:** Changes of inflammatory cytokines in the NIE and IE groups.

**Parameter (pg/ml)**	**IE group**	**NIE group**	***P*-value**
IL-6	9.22 ± 2.06	18.08 ± 3.74	0.04
IL-8	74.81 ± 7.32	101.1 ± 6.00	0.01
IL-10	72.22 ± 5.03	107.7 ± 12.89	0.01
TNF-α	70.88 ± 3.50	96.22 ± 9.50	0.02
IL-1β	47.33 ± 14.47	751.9 ± 48.4	0.0001
IFN-γ	144.8 ± 5.27	112.5 ± 8.55	0.003
IL-6/IL-10	0.10 ± 0.02	0.19 ± 0.03	0.018
IL-8/IL-10	0.85 ± 0.10	1.15 ± 0.10	0.04
TNF-α/IL-10	0.83 ± 0.06	1.20 ± 0.14	0.03
IL-1β/IL-10	0.92 ± 0.38	8.05 ± 0.77	0.0001

*Values are presented as mean ± standard deviation*.

*IL, Interleukin; IFN-γ, interferon-γ; TNF-α, tumor necrosis factor-α; NIE, non-immune-enhancing; IE, immune-enhancing*.

### Clinical Outcome

The incidence of feeding intolerance and the use of sedative drugs were lower in the IE than those in the NIE group, and there were no significant differences between the two groups in terms of mechanical ventilation time, ICU admission time, and length of hospital stay (Table 4). Glutamyl transpeptidase was significantly higher in the NIE feeding than that in the IE group (*P* < 0.05). However, IE feeding did not affect the total protein, prealbumin, and albumin levels (Supplementary Table 3). IE had a positive effect on feeding tolerance.

**Table 4:** Clinical outcome features after nutrition.

**Parameter**		**IE group**	**NIE group**	***P*-value**
Drug	Gastrointestinal motility drugs	15	21	0.26
	Gastrointestinal inhibitory drugs	2	4	0.47
	Tranquilizer	18	11	0.02
	Vasoactive drugs	6	5	0.60
Feeding intolerance	Diarrhea	2	4	0.04
	Flatulence	1	6	
	Gastric retention	1	2	
	ICU stay (days)	23.22 ± 9.67	23.30 ± 10.66	0.98
	Mechanical Ventilation (days)	6.52 ± 7.58	5.23 ± 7.72	0.53
	Hospital stay (days)	39.70 ± 17.81	43.70 ± 24.72	0.49

*Values are presented as mean ± standard deviation*.

*NIE, non-immune-enhancing; IE, immune-enhancing*.

## Discussion

This study shows that early IE is safe and well-tolerated, it can improve the cellular immunity and reduce the serum levels of proinflammatory cytokines. However, we found no evidence regarding the effects of early immune nutrition on the short-term outcomes of patients with severe neurological diseases. Although this is the first trial (according to our PubMed search) to study immune nutrition in patients with severe neurological diseases, it was not surprising that the expected results were not obtained because of the small size and short duration of the study.

Patients in ICUs usually experience excessive mental stress due to pain and anxiety, which leads to a high catabolic and hyper-metabolic state (21, 22). Most clinical studies have confirmed that enteral nutrition is superior to parenteral nutrition (23, 24), and poor feeding tolerance is considered one of the main disadvantages of enteral feeding in critically ill patients. Our results were similar to those reported by Qiu et al. (25): IE nutrition containing medium chain triglycerides, carnitine, and taurine had a lower incidence of feeding intolerance than that of NIE. However, because of the short observation time, we did not observe whether IE had an advantage over NIE nutrition in improving protein intake and the clinical outcomes in patients with severe neurological diseases.

At present, the clinicians' nutritional goals for critically ill patients are to provide energy and meet the patients' needs to restore optimal metabolism and immune response (26, 27). IE nutrition can meet the needs for calorie and protein intake, reduce the occurrence of gastrointestinal intolerance, and regulate the immune effect of patients with severe neurological diseases (3, 28). However, there are limited studies based on the effect of IE nutrition on the immune function in patients with severe neurological diseases after surgery.

In many studies, immune cells, such as T, B, and NK cells have been reduced in patients after surgery or in those with severe diseases, representing a state of suppressed immune function (20, 29). There is increasing evidence that there is a positive correlation between low immune function and high morbidity and mortality (30, 31). Several meta-analyses have reported that the addition of immunomodulatory nutrients to enteral nutrition can significantly reduce the incidence of infectious diseases and promote the recovery of critically ill patients compared with standard enteral nutrition (9, 32). We found that the CD4^+^ T-lymphocyte count and the CD4^+^/CD8^+^ ratio in the peripheral blood were higher in the IE than those in the NIE group. The upregulation of CD4^+^/CD8^+^ can enhance the cellular immune function and promote B-lymphocyte activation and differentiation (33–35). After activation of B-lymphocytes, the secretion of IgM, IgG, and IgA increases, which can enhance humoral immunity (36). This is the theoretical basis for clinical application of immunotherapy.

Costimulatory and co-suppressor molecules are required to participate in the process of T-lymphocyte activation, differentiation, and function (37). CD28 can promote the activation of immune cells and then exert physiological functions, which belong to costimulatory molecules (35). In contrast, TIM-3 and PD-1 are co-suppressor molecules that inhibit effector cells from functioning (38). As the co-suppressor molecules present higher affinity for the ligand than the costimulatory molecules, they have a stronger binding force to the ligand, leading to the inhibition or exhaustion of T-lymphocyte function (39). The surface of fully activated immune cells could also overexpress the inhibitory receptors, such as PD-1 and TIM-3 (40, 41). Our research showed that CD4^+^ and CD8^+^ T-lymphocytes have high CD28 expression, indicating a tendency to induce immune reconstitution and activation. However, there was no significant change in the expression level of inhibitory receptors, probably due to the short detection interval (35, 42).

The results of this study showed that serum IFN-γ levels were higher and TNF-α, IL-6, IL-8, IL-β, and IL-10 levels were lower in patients receiving the immunomodulatory nutrient enteral nutrition than in those receiving standard enteral nutrition. In addition, we observed a higher ratio of pro-inflammatory to anti-inflammatory cytokines in the NIE group. In recent years, the ratio of proinflammatory cytokine IL-6 to anti-inflammatory cytokine IL-10 has been used as a reliable marker for measuring inflammatory status (43, 44). IE nutrition has been shown to reduce inflammatory cytokine levels (45). In systemic diseases caused by inflammation, the expression levels of IFN-γ are inversely related to IL-10 (46–48). Pro-inflammatory factors, such as TNF-α, IL-6, and IL-8, mediate the progression of various diseases and inflammatory responses following brain injury, and are associated with poor prognosis in patients (49–51). Our study found that the difference between the admission and discharge scores on the Glasgow Coma Scale was higher in the IE than that in the NIE group. IE nutrition may have advantages in promoting the recovery of patients' immune function following brain injury. Due to the short observation time in this study, we failed to observe a significant change in the patients' consciousness with the addition of immunomodulatory nutrients to enteral nutrition.

In this study, no improvement was observed in the outcomes of patients with severe neurological diseases, including the ICU length of stay, hospital stay, and 28 days without mechanical ventilation because of IE nutrition. A possible explanation is that short-term enteral nutrition may not be sufficient to affect patient outcomes. Future researches may require larger sample sizes and longer research duration to confirm whether IE has beneficial effects on patients with severe neurological diseases.

In summary, enteral nutrition with taurine, L-carnitine, and medium chain triglycerides is safe and well-tolerated, and it can improve the cellular immunity and modulate inflammatory reaction in patients with severe neurological diseases.

## Data Availability Statement

The original contributions presented in the study are included in the article/Supplementary Materials, further inquiries can be directed to the corresponding author.

## Ethics Statement

The studies involving human participants were reviewed and approved by Research and Clinical Trial Ethics Committee of the First Affiliated Hospital of Zhengzhou University. The patients/participants provided their written informed consent to participate in this study. Written informed consent was obtained from the individual(s) for the publication of any potentially identifiable images or data included in this article.

## Author Contributions

DW and KC participated in the research design and coordination and helped to draft the manuscript, and conducted the experiments. NM and QL contributed the clinical sample collection. XS, HY, and RS performed the data analysis. All authors read and approved the final manuscript.

## Conflict of Interest

The authors declare that the research was conducted in the absence of any commercial or financial relationships that could be construed as a potential conflict of interest.

## Publisher's Note

All claims expressed in this article are solely those of the authors and do not necessarily represent those of their affiliated organizations, or those of the publisher, the editors and the reviewers. Any product that may be evaluated in this article, or claim that may be made by its manufacturer, is not guaranteed or endorsed by the publisher.

## Abbreviations

APACHE: Acute Physiology and Chronic Health Evaluation
GCS: Glasgow Comma Scale
IE: immune-enhancing
ICU: intensive care unit
IL: interleukin
NIE: non-immune-enhancing
SICU: Surgical Intensive Care Unit
TNF: tumor necrosis factor.
