# Supplementary material for: Beneficial Effect of Immune-Enhanced Enteral Nutrition on Immune Function in Patients With Severe Neurological Diseases: A Single-Center Randomized Controlled Trial
Source: Front Nutr. 2021 Aug 23;8:685422. doi: 10.3389/fnut.2021.685422 (PMC8419436; doi:10.3389/fnut.2021.685422)
Supplement: Supplementary file 1 [file Data_Sheet_1.pdf]

Supplementary table 1. Enteral nutrition ingredients in the IE and NIE Groups.

component	unit	IE group	NIE group
Energy density	kcal/ml	1.05	1.00
Protein	calorie %	15.0	14.2
Carbohydrate	calorie%	56	54
Lactose	g/ml	0	0
Fat	calorie %	29.0(LCT 80%,MCT 20%)	31.8（LCT 100%）
Omega-3	g/ml	0.09/100	0/100
Omega-6	g/ml	0.62/100	1.94/100
Taurine	mg/ml	10/100	0
L-carnitine	mg/ml	8.4/100	0
Dietary fiber	g/ml	1.06/100	0

LCT, Long chain triglycerides；MCT， medium chain fatty acid; NIE, non-immune-enhancing; IE immune-enhancing.

Supplementary table 2. Simple gastrointestinal function assessment.

**Content**	**Scoring**			
Score	0	1	2	5
Flatulence / Bellyache	None	Mild bloating without abdominal pain	Obvious abdominal distension or abdominal pain relieves itself or intra-abdominal pressure 15 ~ 20mmHg	Severe abdominal distension or abdominal pain cannot relieve itself or intra-abdominal pressure> 20mmHg
Nausea / Vomit	None， or Continuous gastric decompression asymptomatic	Nausea but no vomiting	Nausea and vomiting (without gastrointestinal decompression) or GRV> 250 ml	Vomiting with gastrointestinal decompression or GRV> 500 ml
Diarrhea	None	Thin stool 3-5 times / d and the amount is less than 500ml	Thin stool ⩾5 times / d and 500-1500ml	Thin stools ⩾5 times / d and the amount ⩾1500ml

Supplementary table 3. Nutrition States and Liver Function in the IE and NIE Groups.

Parameter	Feed	D0	D6	D
	IE	22.23±2.24 [a]	22.81±2.12	23.32±3.52
BMI, kg/m^2	NIE	22.43±2.58	22.73±3.24	23.51±2.82
	P value	NS	NS	NS
	IE	65.41±9.78	63.56±7.40	65.01±6.83
TP, g/L	NIE	65.28±9.53	62.58±6.20	65.12±5.82
	P value	NS	NS	NS
	IE	35.37±7.32	36.09±5.22	36.34±5.10
ALB, g/L	NIE	35.08±7.26	34.62±3.97	36.32±4.28
	P value	NS	NS	NS
	IE	173.07±56.94	191.07±65.97	200.85±56.08
PA, g/L	NIE	186.93±55.98	189.57±60.85	222.97±62.14
	P value	NS	NS	NS
	IE	40.20(58.90)[b]	57.00(94.00)	43.00(34.25)
ALT, U/L	NIE	36.80(38.95)	59.50(122.25)	37.00(40.50)
	P value	NS	NS	NS
	IE	47.10(64.10)	44.00(41.00)	36.50(17.75)
AST, U/L	NIE	44.70(39.98)	45.00(64.00)	29.50(12.75)
	P value	NS	NS	NS
	IE	14.40(14.66)	11.32(8.29)	7.30(9.13)
TBIL, μmol/L	NIE	15.00(15.78)	8.62(6.82)	8.26(3.98)
	P value	NS	NS	NS
	IE	5.90(9.40)	4.46(4.01)	5.17(4.71)
DBIL, μmol/L	NIE	6.30(8.62)	3.87(4.01)	3.72(3.05)
	P value	NS	NS	NS
	IE	7.10(5.90)	5.40(5.20)	4.20(3.70)
IBIL, μmol/L	NIE	8.50(5.30)	4.40(3.18)	3.50(2.65)
	P value	NS	NS	NS
	IE	50.00(98.80)	87.00(87.00)	44.00(50.75)
GGT, IU/L	NIE	50.50(73.25)	94.50(110.25)	95.00(103.75)
	P value	NS	NS	0.03
	IE	101.60(93.40)	140.00(93.00)	112.50(53.75)
ALP, U/L	NIE	112.50(39.10)	105.50(41.50)	116.00(44.00)
	P value	NS	NS	NS
	IE	68.30(93.54)	29.83(90.10)	15.11(18.02)
CRP, mg/L	NIE	48.73(46.60)	26.40(43.89)	10.50(18.15)
	P value	NS	NS	NS
	IE	0.35(0.92)	0.18(0.39)	0.13(0.39)
PCT, ng/mL	NIE	0.30(1.02)	0.18(0.28)	0.11(0.16)
	P value	NS	NS	NS
	IE	13.51±5.06	12.09±4.88	8.87±3.08
WBC, 10^9/L	NIE	11.67±3.63	13.67±4.91	8.35±2.64

P value	NS	NS	NS

BMI, body mass index; TP, total bilirubin; ALB, albumin; PA, prealbumin; ALT, alanine aminotransferase; AST, aspartate aminotransferase; TBIL, total bilirubin; DBIL, direct bilirubin; IBIL, indirect bilirubin; GGT, glutamyl transpeptidase; ALP, alkaline phosphatase; CRP, C-reactive protein; PCT, procalcitonin; NS, not significant; D, Examination results on the day of discharge; NIE, non-immune-enhancing; IE immune-enhancing; WBC, White blood cell count.

[a] Values are mean ± standard deviation. [b] Values are Median (interquartile range)
