# Supplementary figures and images for: Beneficial Effect of Immune-Enhanced Enteral Nutrition on Immune Function in Patients With Severe Neurological Diseases: A Single-Center Randomized Controlled Trial
Source: Front Nutr. 2021 Aug 23;8:685422. doi: 10.3389/fnut.2021.685422 (PMC8419436; doi:10.3389/fnut.2021.685422)

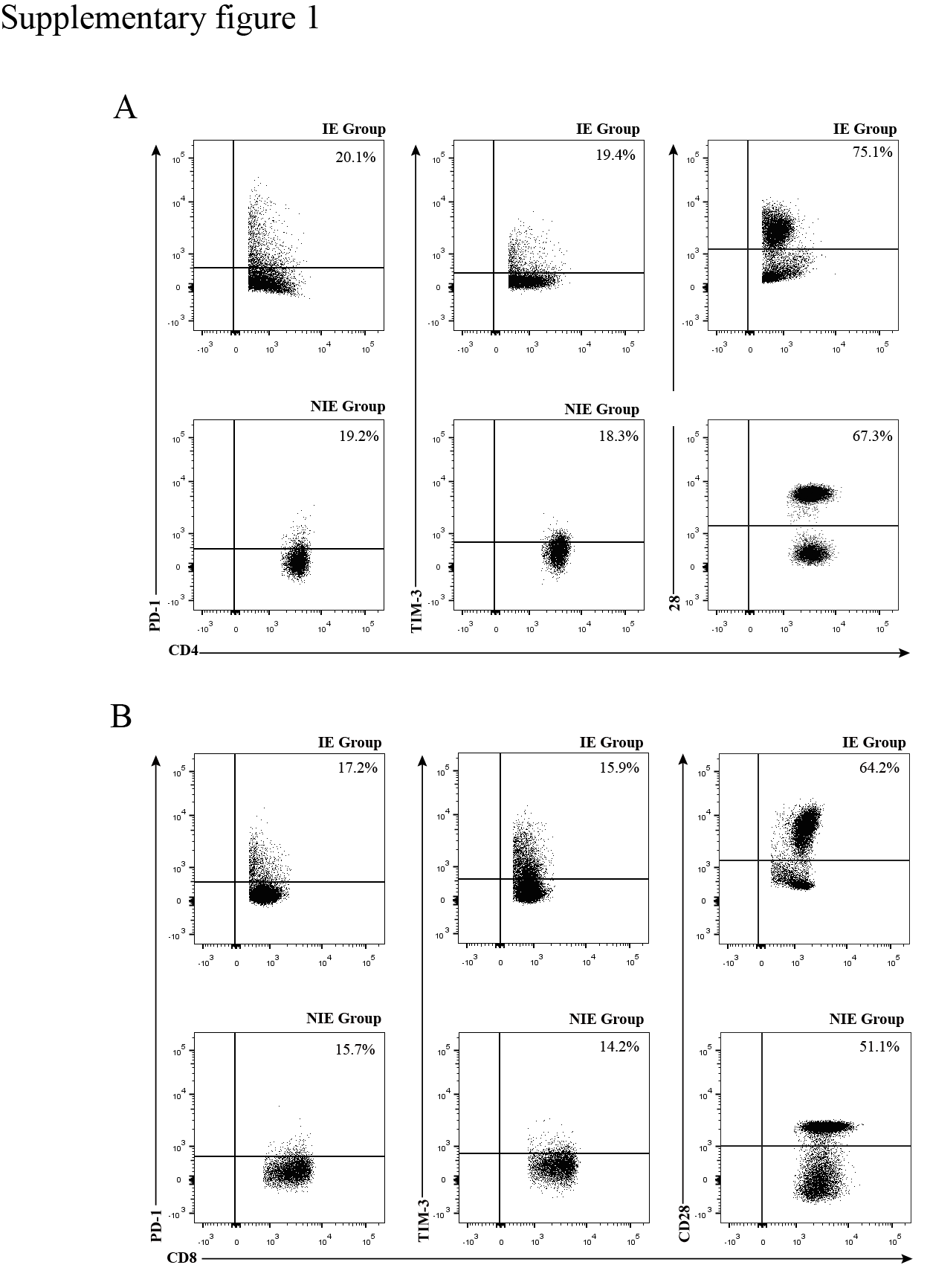

Supplement: Supplementary file 2 [file Image_1.TIF]
